# When philosophical nuance matters: safeguarding consciousness research from restrictive assumptions

**DOI:** 10.3389/fpsyg.2023.1306023

**Published:** 2023-11-27

**Authors:** Marius Usher, Niccolò Negro, Hilla Jacobson, Naotsugu Tsuchiya

**Affiliations:** ^1^School of Psychological Sciences, Tel Aviv University, Tel Aviv, Israel; ^2^Sagol School of Neuroscience, Tel Aviv University, Tel Aviv, Israel; ^3^Monash Centre for Consciousness and Contemplative Studies, Monash University, Melbourne, VIC, Australia; ^4^Department of Cognitive and Brain Sciences, Hebrew University of Jerusalem, Jerusalem, Israel; ^5^Department of Philosophy, Hebrew University of Jerusalem, Israel; ^6^Faculty of Medicine, Nursing, and Health Sciences, School of Psychological Sciences, Monash University, Melbourne, VIC, Australia; ^7^Laboratory of Qualia Structure, ATR Computational Neuroscience Laboratories, Hikaridai, Seika-cho, Soraku-gun, Kyoto, Japan

**Keywords:** consciousness, unfolding argument, functionalism, recurrency, causal structure, IIT, Blockhead

## Abstract

In this paper, we revisit the debate surrounding the Unfolding Argument (UA) against causal structure theories of consciousness (as well as the hard-criteria research program it prescribes), using it as a platform for discussing theoretical and methodological issues in consciousness research. Causal structure theories assert that consciousness depends on a particular causal structure of the brain. Our claim is that some of the assumptions fueling the UA are not warranted, and therefore we should reject the methodology for consciousness science that the UA prescribes. First, we briefly survey the most popular philosophical positions in consciousness science, namely physicalism and functionalism. We discuss the relations between these positions and the behaviorist methodology that the UA assumptions express, despite the contrary claim of its proponents. Second, we argue that the same reasoning that the UA applies against causal structure theories can be applied to functionalist approaches, thus proving too much and deeming as unscientific a whole range of (non-causal structure) theories. Since this is overly restrictive and fits poorly with common practice in cognitive neuroscience, we suggest that the reasoning of the UA must be flawed. Third, we assess its philosophical assumptions, which express a restrictive methodology, and conclude that there are reasons to reject them. Finally, we propose a more inclusive methodology for consciousness science, that includes neural, behavioral, and phenomenological evidence (provided by the first-person perspective) without which consciousness science could not even start. Then, we extend this discussion to the scope of consciousness science, and conclude that theories of consciousness should be tested and evaluated on humans, and not on systems considerably different from us. Rather than restricting the methodology of consciousness science, we should, at this point, restrict the range of systems upon which it is supposed to be built.

## 1 Introduction

Understanding how consciousness relates to the structure and activity of the brain is one of the most challenging tasks of scientific endeavor. Whereas a few decades ago, the subject of consciousness was exclusively philosophical, it has become a major subject of research in neuroscience in the last few decades. What started as a search for neural correlates of consciousness (NCC) (Crick and Koch, 1990) - for a discussion, see (Chalmers, 2000) - has now matured to the development of a multitude of theories that aim to answer the more difficult question of how consciousness can be explained by the organization of brain processes (Dehaene et al., 1998; Lamme, 2006; Tononi et al., 2016; Solms and Friston, 2018; Solms, 2019; Gidon et al., 2022; Seth and Bayne, 2022). These theories are subject to an intensive debate that involves experimental research (Crick and Koch, 1998; Zeki and Bartels, 1998; Landman et al., 2003; Sligte et al., 2008; Aru et al., 2012; de Graaf et al., 2012; Liu et al., 2012; Bronfman et al., 2014; King and Dehaene, 2014; Mudrik et al., 2014; Noy et al., 2015; Josselyn and Tonegawa, 2020; He, 2023), philosophical analysis (Block, 1995, 2011; Chalmers, 1995, 1996; Phillips, 2011, 2016; Cohen et al., 2016; Usher et al., 2018; Bronfman et al., 2019; Ellia et al., 2021; Ellia and Chis-Ciure, 2022; Michel, 2023), as well as clinical/neuropsychological research (Owen et al., 2006; Monti, 2015). These aspects of consciousness research are necessarily intertwined, because all the consciousness theories have specific philosophical starting points and implications.

This interplay between abstract theoretical considerations and experimental research is illustrated by a recent philosophical argument – the *unfolding argument* (UA) – which has been proposed with the aim of prescribing which types of consciousness theories are scientifically valid, prior to empirical testing (Doerig et al., 2019). According to the UA, causal-structure theories, such as the *Integrated Information Theory* (IIT) (Tononi et al., 2016) and the *Recurrent Processing Theory* (RPT) (Lamme, 2006), are either false or unscientific. This reduction of the theory space could be beneficial for consciousness research, as there is currently a proliferation of consciousness theories (for discussions, see Aru et al., 2020; McFadden, 2020; Del Pin et al., 2021; Doerig et al., 2021; Signorelli et al., 2021; Seth and Bayne, 2022). However, the UA also has its own philosophical assumptions, which have come under severe criticism (Kleiner, 2020; Negro, 2020; Tsuchiya et al., 2020; Albantakis, 2020a; Kent and Wittmann, 2021; Kleiner and Hoel, 2021; Mallatt, 2021; Usher, 2021). The authors of the UA have responded to this criticism (Herzog et al., 2022). More recently, Doerig et al. (2021) have expanded the UA into a research program that is meant to set up a set of “hard criteria for empirical theories of consciousness,” which not only restricts the type of admissible consciousness theories, but also explicitly prescribes a restrictive methodology for consciousness research.

The aim of this paper is to argue that the UA research program is too restrictive, making explicit the specific points of disagreement, and more generally to show how implicit assumptions can influence our methodological choices in consciousness science. By bringing to the fore a variety of implicit assumptions and reconsidering the relations of extant scientific theories to traditional philosophical positions and arguments regarding the nature of consciousness and the feasibility of its scientific investigation, we hope to make the debate more informative. This is important, given the high price of prematurely abandoning promising classes of theories without empirical testing (see (Melloni et al., 2021, 2023) for promising attempts to test consciousness theories). In doing so, we aim to broaden the discussion to several central issues that are critical to consciousness research, such as (i) the grounding of consciousness theories in functionalism, mind-brain (MB) type-identity, or behaviorism; and (ii) the reality of phenomenal experience and the role of first-person and neural evidence in consciousness science. Finally, we aim to propose an alternative research program for consciousness research, which is bold in its methodology, but somewhat more restrictive in its scope (to account for human consciousness, first). We start with a brief recap of the broad philosophical positions on consciousness and a summary of the UA before we critically examine its soundness.

## 2 Philosophical positions in consciousness research

Given that many concepts employed in the contemporary science of consciousness derive from philosophy, it is important to have a clear understanding of some influential philosophical frameworks on consciousness. Historically, in Western philosophy the traditional, theory on the relation between mind and matter was *dualism*, the view that mind and matter are fundamentally distinct (Descartes, 1641/1996). There are various versions of dualism, but they are not popular in contemporary consciousness research.^1^ Scholars in this field mostly follow *physicalism* (Francken et al., 2022; Bourget and Chalmers, 2023) – the view that consciousness supervenes with metaphysical necessity on the physical.

There are many ways to make the mind–body relation more precise, under a physicalist framework. A first option is behaviorism, which considers the mind as a set of behavioral dispositions. Historically, behaviorism has been presented either as logical behaviorism, the view that mental terms can be conceptually reduced, via *a priori* analysis, to behavioral terms (Ryle, 1949), or as methodological behaviorism, which was motivated by the drive to base psychology on firm scientific grounds by focusing on purely outer and publicly observable phenomena (Watson, 1913); reprinted in Watson (1994). Despite being quite influential in the past, behaviorism is now widely accepted to be deficient as an account of mental states and processes [see textbook discussions in (Braddon-Mitchell and Jackson, 2007) and (Bayne, 2021)]. We thus focus on two more influential physicalist theories, which both play a central role in the debate surrounding the UA against causal-structure theories: (i) the *mind*-*brain (MB) type identity theory* (Place, 1956; Smart, 1959); and (ii) *functionalism* (Putnam, 1967).

1. **MB-type-identity.** According to type-identity theorists, types of mental states are types of physical (brain) states, in the same way as *water is H_2_O* (or *clouds are vapor*; Place, 1956; Smart, 1959). Conscious states such as pain are thought to be identical to specific types of brain states (e.g., a particular type of *cortical-thalamic neural oscillation*). According to the type-identity theory, the identity of conscious mental states is determined by their physical constitution.
2. **Functionalism**. Originally, functionalism has been developed in opposition to both MB-identity theory and behaviorism, and was motivated by the *multiple realizability* argument (Putnam, 1967), which asserted that it is unlikely that all mental states (or processes) of the same type (e.g., pain or the desire to drink water) are always realized, and moreover *must* be realized, by the same type of brain states (or processes). According to *functionalism,* it is not the material constitution of mental states that determines their identity; instead, it is the role they play in the cognitive system of which they are a part. This independence of functional roles from their substrate can be expressed in different ways. A distinction, which will play a role in our later discussion, can be drawn between the material properties of the substrate (e.g., whether it is made of carbon or silicon) and the structural properties of the substrate (e.g., its *network* connectivity). A theory can be substrate-independent with respect to the material properties of the substrate without being independent with respect to its structural/network properties. More specifically, according to functionalism, the identity of a mental state, such as pain, is determined by its causal relations to sensory inputs, behavioral outputs, and, importantly, to *other mental states*. This focus on the relations among internal mental states marks the main difference between *functionalism* and behaviorism, which conceives of mental properties as behavioral dispositions (Fodor, 1981; Braddon-Mitchell and Jackson, 2007) independent of transitions among internal states that mediate between stimulus and responses. This difference between functionalism and behaviorism is eloquently illustrated in a review by Fodor (1981), portraying the main difference as follows: “According to logical behaviorism,^2^ it is a necessary truth that any system that has our stimulus–response contingencies also has our headaches” (Fodor, 1981, p. 118). For functionalism, on the other hand, mental states (e.g., headaches) are determined by their place in the cognitive algorithm that generates the stimulus–response contingencies. This can be applied to phenomenal experiences: for a functionalist, the phenomenal character of a mental state depends on the cognitive algorithm in which that state plays a role; that is, consciousness is reducible to the functional and relational profile a mental state bears to stimuli, behavior, and other mental states. Although there are many versions of functionalism (see (Braddon-Mitchell and Jackson, 2007) for textbook discussion), this introduction will be enough for the present purposes.

Consciousness is considered to pose a problem for all physicalist theories (see, e.g., Jackson, 1982), but there are also special difficulties that it is thought to pose for functionalism. In particular, while functionalism has been seen as a very successful approach in cognitive research^3^ (Block and Fodor, 1972), it has been attacked as an account to consciousness by two lines of argument, which stem from the idea that phenomenal properties are *intrinsic* properties, and cannot be fully captured by the *relational,* functional, properties functionalism focuses on. A prominent example of this line of challenge is provided by the inverted-spectrum (or qualia) arguments (Block and Fodor, 1972; Shoemaker, 1975, 1982; Palmer, 1999), which aim to show that two systems can have mental networks with the same functional profile, while having different (more precisely, inverted) experiences, or the converse (Block, 1990). A second anti-functionalist objection is *the absent qualia argument* (Block, 1978). Perhaps one of the sharpest attacks on functionalism as a theory of consciousness (belonging to the second class of anti-functionalist objections) is Ned Block’s *China-Brain* (Block, 1978), which asks us to consider a simulation of a human brain, in which all neurons in that brain are replaced by a large set of people (he offers the population of China to the task), with all communication between the neurons replaced by telephone communication between the people in this population. Supposing the China population is linked to sensory and motor organs in the same way as the original person’s brain, this (China-brain) simulation will produce the same behavior as a normal person (perhaps in slow motion).

The intuition that Block appeals to is that while we readily accept that the original person has, e.g., gustatory phenomenal experiences, when she consumes a chocolate ice cream, we feel reluctant to accept the same for the China population. While this argument is not conclusive – it has not persuaded most functionalists (see (Braddon-Mitchell and Jackson, 2007) for a textbook discussion), who can insist that the simulation (i.e., the population of China in this example) has the same experiences as the simulated person – we wish to mark this as a central argument, since, as we will see, it has much in common with the UA, to which we turn next.

## 3 The unfolding argument

The unfolding argument (UA) was proposed to refute a large set of consciousness theories called *causal-structure* theories^4^, namely theories that hold that consciousness depends on the causal structure of the brain. Two prominent theories of this sort are the integrated information theory (*IIT*) (Oizumi et al., 2014; Tononi and Koch, 2015; Albantakis et al., 2023) and the recurrent processing theory (*RPT*) (Lamme, 2006), which both assume that consciousness depends on the presence of recurrent brain connectivity. Categorizing IIT and RPT in relation to traditional philosophical positions is not straightforward (Tononi and Koch, 2015; Tononi, 2017; Grasso, 2019; Cea, 2020; Negro, 2022; Tononi et al., 2022). According to both theories, consciousness depends on the causal structure of the brain, but both theories hold that consciousness may be multiply realizable, and allow that it can be realized in non-biological systems, as long as those systems have the *abstract* (i.e., independent of the specific and fine-grain biological details) network-structure that the theories associate with consciousness. This alignment with multiple realizability may suggest that IIT and RPT are compatible with functionalism^5^. Interestingly, despite the UA-proponents arguing to endorse a functionalist approach, they still consider these models as false or outside the range of science. This seems to be because UA proponents appear to consider functional characteristics at a lower level of resolution (i.e., less sensitive to specific properties of the system), namely at the behavioral rather than at a network level (which is already relatively abstract). For example, a functional characteristic, such as network-recurrency, which IIT and RPT assume necessary for consciousness, is considered by the UA-proponents as an *implementation* detail that can be multiply realized by a system without network-recurrency (i.e., a feedforward network), as long as the latter is behaviorally equivalent to the original (recurrent network) in consciousness experiments. We thus come to a somewhat paradoxical situation, in which an argument that is framed to support functionalism, rules out (as false or outside the range of science), on purely theoretical grounds, a class of models that could be compatible with functionalism under some interpretations. In the next section we shall examine whether the UA supports, or even coheres with functionalism in general.

In particular, the UA proponents claim that functional properties can be behaviorally measured from the third-person perspective without assuming that a particular brain architecture (e.g., recurrent vs. feedforward) determine consciousness in the first place, and therefore only a theory that associates consciousness with functional/behavioral properties can be confirmed or falsified through behavioral research. In particular, they assert that “consciousness must be described in terms of what it does, and not how it does it” (Doerig et al., 2019, p. 56). According to the UA, for any conscious (recurrent) brain that mediates behavior in a consciousness experiment, we can construct a brain that replaces the recurrent (RN) with feed-forward networks (FFN), that is behaviorally equivalent to the original brain. Therefore, as no behavioral experiment testing consciousness can distinguish between such brain variants, the UA concludes that all theories that assume consciousness to depend on a certain causal structure (e.g., recurrent vs. feed-forward) are either false or unfalsifiable.

The UA has the following form:

“(P1): In science we rely on physical measurements (based on subjective reports about consciousness).

(P2): For any recurrent system with a given input–output function, there exist feedforward systems with the same input–output function (and vice-versa).

(P3): Two systems that have identical input–output functions cannot be distinguished by any experiment that relies on a physical measurement (other than a measurement of brain activity itself or of other internal workings of the system).

(P4): We cannot use measures of brain activity as a-priori indicators of consciousness, because the brain basis of consciousness is what we are trying to understand in the first place.

(C): Therefore, EITHER causal structure theories are falsified (if they accept that unfolded, feedforward networks can be conscious), OR causal structure theories are outside the realm of scientific inquiry (if they maintain that unfolded feedforward networks are not conscious despite being empirically indistinguishable from functionally equivalent recurrent networks)” (Doerig et al., 2019, p. 53).

This conclusion rules out causal-structure theories from consciousness science, without the need to test them on their ability to account for data. A further UA-variant has been proposed, which replaces P2 (the behavioral equivalence between RN and FFN) with the behavioral equivalence between a physical system and its computer simulation (Herzog et al., 2022). Accordingly, instead of building a behaviorally equivalent FF-robot, we can create a robot controlled by a computer simulation of a real brain. Herzog et al. (2022) argue that, since we are typically running our computer simulations of RNs on serial computers, such a robot will be indistinguishable from the actual person, regarding any consciousness test. And therefore, we would not have any reason to deem one system as conscious and the other as non-conscious.

We believe this conclusion is premature and that both variants of the UA are unsound. Still, before we turn to our counterarguments, we wish to note that the simulation version is similar to the simulation created by Block’s China-Brain argument in invoking two functionally identical systems in order to show, on *a priori* grounds, that a particular approach to consciousness is invalid. Yet, the two arguments suggest opposite conclusions – the UA was intended to cohere with functionalist approaches, whereas the China-Brain was intended to undermine functionalism. Even if the China-Brain argument is inconclusive, we believe it would be surprising if this opposite argument were accepted as conclusive.

To better understand this complex dialectic, we must focus on the exact relation between the UA and functionalism. In the next section, we point out that the UA-rationale can lead to a stronger argument that rules out as invalid not only causal-structure scientific theories of consciousness, but also (and perhaps contrary to the original motivation) functionalist theories.

## 4 An UA-type argument against functionalism (and cognitive science)

In section two, we have surveyed the essential distinctions between functionalism and behaviorism. In their response to critics, Herzog et al. (2022) have clarified that their position is *functionalist* rather than *behaviorist.*^6^ We accept that they are motivated to account for consciousness based on latent processes of the organism and that this is consistent with functionalism, which is a productive framework in cognitive science. However, we believe that some of the assumptions of the UA are not consistent with functionalism, but rather point in a direction closer to behaviorism. This is because (as we will shortly argue, in subsections 4.1 and 4.2) these assumptions together with the basic rationale of the argument and a plausible assumption regarding the indeterminacy of algorithm by behavior lead to the conclusion that theories according to which consciousness is determined by the system’s algorithms, or information processing, are just as problematic as causal structure theories. According to behaviorism, mental properties are exclusively determined by behavioral dispositions, while according to functionalism, mental properties are determined by the algorithm that underlies behavior (Putnam, 1967; Fodor, 1981). Furthermore, functionalism regards the causal relations among the system’s internal mental states as crucial to their identity, which is closer to the causal structural theories’ claim. This dissociation between behavior and cognitive algorithm was clearly illustrated in the *Blockhead-*argument (Block, 1981), in which we are provided with a dissociation between behavior and algorithm.

The argument compares a person who acts as a result of information processing with a robot, called Blockhead, that acts the same as a person, as a result of inspecting a large look-up table that contains an extensive list of behaviors that ordinary people are likely to provide in response to possible questions (Block, 1981). Functionalists have accepted this dissociation, as an illustration of a functionalist thesis that Block calls *psychologism*, according to which mental properties such as intelligence depend on the character of the internal information processing that produces the relevant behavior and not on the input–output behavior alone. As formulated by Block, *psychologism* is the doctrine that:

"Two systems could have actual and potential behavior typical of familiar intelligent beings, that the two systems could be exactly alike in their actual and potential behavior, and in their behavioral dispositions and capacities and counterfactual behavioral properties (*i.e.,*, what behaviors, behavioral dispositions, and behavioral capacities they would have exhibited had their stimuli differed) – the two systems could be alike in all these ways, yet there could be a difference in the information processing that mediates their stimuli and responses that determines that one is not at all intelligent while the other is fully intelligent" (Block, 1981, p. 5).

Critically, we will argue that insisting that the only available resource in trying to account for consciousness is input–output behavior (P3-P4) conflicts with this principle and seems more consistent with *behaviorism* than with *functionalism*. At a first pass, the position of UA proponents brings to mind *methodological* behaviorism and is silent about accounts of the metaphysics of consciousness, since it is possible to hold that consciousness is constituted by the functional profile of a physical system while maintaining that the functional profile itself can be detected by looking only at behavioral responses. However, we will attempt to show that (at least in conjunction with the other premises of the UA) the methodology the UA prescribes, leads to the conclusion that both MB type-identity theories and functionalist theories lie outside the realm of scientific inquiry. If this is the case, then in present context, the methodological thesis (P1, P3-4) implies that no metaphysical theory that attempts to uncover the internal underpinning (whether neural or functional) of external input–output patterns is within our reach. We argue that this way of addressing mental phenomena is an overly austere (and restrictive) methodology that ignores actual practice in the cognitive sciences.

A similar analysis of the UA argument has been recently presented by Kleiner (2020); see also (Kleiner and Hoel, 2021), who concluded that on the basis of the UA premises one could rule out (as false or unscientific) any functionalist theory of consciousness^7^, including theories such as the Global-Workspace (Dehaene et al., 1998). This is because (following the UA rationale), one cannot distinguish (on the basis of input–output functions) between a system that is driven by a global-workspace and one driven by a lookup table of it. In their reply to Kleiner (2020), Herzog et al. (2022) argue that:

“if the workspace is defined in functional terms, then the lookup table also realizes a global workspace. Contrary to causal structure, there is no mathematical theorem stating that the same i/o functions can be realized with and without a global workspace (see also Ganesh, 2020). In summary, we agree that Kleiner’s argument applies to theories that identify consciousness with a certain non-functional process claimed to be necessary and sufficient as, indeed, many theories do (Doerig et al., 2021). However, theories may be cast in functional terms, or propose that consciousness should not simply be identified with a single process, just as life is not identified with a single process (Machery, 2012).” (Herzog et al., 2022, p. 10).

We believe that there is an important ambiguity in this statement that is critical to the differences between behaviorism and functionalism. Indeed, functionalism allows for the possibility that mental properties (e.g., pains) do not uniquely determine the neural structure or processes. However, it still requires them to be uniquely associated with the functional algorithm, which generates behavior (Fodor, 1981). The question is in what sense the global workspace and its look-up table are functionally identical? If the difference between these two systems is to be found at the neural level, one may suggest (as we interpret Herzog et al. to argue) that we have multiple neural processes that implement the same global-workspace algorithm, all of which are associated with consciousness. However, this functionalist solution becomes out of reach if we have two different *cognitive algorithms* that mediate the same input–output contingency: a global workspace and its lookup table. It is not clear to us on what basis it would be the case that the Global-Workspace and its lookup-table are identical, *qua algorithm*. The problem is that if the global workspace and its lookup table are not identical, *qua* cognitive algorithms, then, at best, they can be equivalent only in terms of outer behavioral dispositions. And if this is the level at which consciousness should be investigated, then a version of behaviorism follows. This result, we believe, would exclude not only causal structure theories from consciousness science, but also functionalist theories. This result is possible only if a dissociation between behavior and the cognitive algorithm were possible. In the next section, we expand on why such an indeterminacy (similar to the one suggested by the UA between behavior and neural structure) is likely to manifest between behavior and cognitive algorithm, under the restrictive methodology advocated by the UA.

### 4.1 The behavior-algorithm indeterminacy

In the previous sub-section, we have highlighted that if multiple cognitive algorithms can determine the same behavior, then a similar rationale as that of the UA could also apply to functionalist theories of consciousness. In this sub-section, we unpack this claim by beginning with the traditional distinction among different levels of describing cognitive systems^8^.

Level 1 (“behavioral level”): Describes the input–output function of the system – i.e., its actual and potential behavior and responses to any possible stimuli. In this context, the inputs and outputs are mathematical values (that can also represent properties in the physical world). The I-O function describes the “problem” to be computed.

Level 2 (“functional-algorithmic level”): Describes the algorithm by means of which the Input–Output function of level 1 is being computed. It describes the specific information processes by means of which the system solves the problem (of how to achieve the outer behavior).

Level 3 (“physical-implementation level”): Describes the physical structure that implements the algorithm of level 2.

Now, return to the UA. The argument crucially appeals to the assumption that the behavioral level does not determine the causal structure, namely the physical implementation level: the exact same I/O function can characterize systems with multiple causal structures. This is *P2* – “for any recurrent system with a given I/O function, there exist feedforward systems with the same input–output function” (Doerig et al., 2019, p. 53) – generalized to all causal structure theories (as it should, if it is to prove its conclusion regarding all causal-structure theories). The proponents of the UA argue that I/O functions provide the primary evidence for scientific theories of consciousness. Assuming this, they conclude that causal structures are not the right place to look for consciousness, if we are after a *scientific* explanation of consciousness. However, just as it can be argued that the behavioral level cannot determine causal structure, so it can be argued that, likewise, it cannot determine the functional-algorithmic level.^9^ In terms of the triple distinction above, level 1 may not determine both *level 3 and level 2*. We thus formulate P2* in this way: each I/O function can be computed by many different algorithms, just as each algorithm can be realized by different physical structures.

The most straightforward illustration of the claim that different algorithms can compute any I/O function is provided by the above-mentioned “Blockhead thought experiment” (Block, 1981). The Blockhead’s and a real person’s algorithms are drastically different, even if they produce the same behaviors - so, they are Level 1-equivalent, but not Level 2-equivalent. The real person carries a variety of cognitive processes (such as mental inferences, goal directed memory search, value-estimations, etc.), while Blockhead only searches its lookup-table and selects the first possible response. The Blockhead system, however, can compute only functions that range over a finite number of input-arguments. Here, then, are other examples that extend this (finite) limitation. The first only illustrates the rationale underlying the (unlimited range) claim.

Take any algorithm that receives an input x and outputs f(x). Start by adding two steps before you start the original algorithm: first, add n to x (Step 1: *y* = x + n). Then subtract n from the result (Step 2: *z* = y-n). You are back at x. Step 3, continue with the original algorithm. Since this can be done with any n, we have infinitely many algorithms for the same I/O function. The second illustration concerns the highly instrumental sorting algorithms – ones that arrange elements of a list in a particular order (e.g., from highest to lowest).^10^ Importantly, there is a mathematical proof that *any* computable function can be computed by different algorithms^11^ (Miller, 2014). This brief discussion substantiates our claim that two systems that are indistinguishable with respect to Level 1 (I/O behavior) can be different with respect to Level 2 (algorithm): behavior is not sufficient for determining cognitive functions [see Albantakis (2020b) for a vivid illustration of this idea, showing that the same behavioral function can be computed by multiple algorithms, each involving a different number of conscious entities].

### 4.2 Upshot: the argument proves too much

The upshot of the reasoning presented above is as follows. If the only available data is behavioral (as characterized by inputs and outputs), then it may be impossible (under restrictive conditions, see below) to differentiate between different physical-implementation theories: there can be multiple theories that explain sensory inputs and behavioral outputs equally well. However, under similarly restrictive conditions, it may also be impossible to differentiate between theories concerning the algorithmic (cognitive) processes that generate the behavior. The indeterminacy of causal structure by input–output functions expressed by the original (P2) may plausibly apply to the level above it (level 2), as in both cases different theories – specifying different physical structures and different algorithms, respectively – can underlie the same input–output functions (P2*). Generalizing the rationale of the UA, not only causal structure theories, but also theories attempting to uncover the specific information processes, algorithms or computations that underlie the input–output functions that characterize familiar conscious systems, would then be deemed invalid or unscientific.^12^ This conclusion is undoubtedly too strong and must be rejected, because it would drastically reduce the number of viable and productive approaches to study consciousness. Given that progress in the field has clearly been made, this is an unwarranted conclusion. Hence, the rationale that leads to it must be rejected. And since this rationale is similar to that employed by the original UA (in deducing that a group of theories is unscientific by appealing to an indeterminacy assumption), the UA itself should be rejected.

Let us be more explicit about why the relevant conclusion is untenable. It directly results (based on purely *a priori* grounds) in a refutation of functionalism as a valid approach to consciousness (see also Kleiner, 2020). The methodology suggested by the UA is thus closer to behaviorism, as grounded on the assumption that consciousness is whatever results in behavior obtained in consciousness experiments. This conclusion (ruling out functionalist theories as non-scientific), implied by the very rationale of the UA and reflected by the methodology its proponents suggest, contradicts the viewpoint of UA proponents themselves. Moreover, it appears that significant progress has been obtained in cognitive science, demonstrating that the indeterminacy between behavior and algorithm, can be resolved by relying on less restrictive methods and thus progressing beyond the “observable data” to infer the underlying entities and processes. Therefore, the UA – or its underlying rationale – proves too much and there must be something wrong with it. In what follows, we point out a few weaknesses in the UA reasoning.

## 5 Examining the philosophical premises of the UA (P1, P3, P4)

### 5.1 The scientific significance of the first-person perspective

We will examine the UA-premises to understand what must go wrong in the argument we just presented. If even one of these premises is false, the UA-conclusion that causal structure theories of consciousness are false or unfalsifiable would be undermined. Similarly, the conclusion that functionalist theories of consciousness are unscientific would be undermined by negating any of the P1, P2*, P3, P4 statements.

Here we focus on the more philosophical (P1, P3, P4) premises, for two reasons. First, while we also reject P2 – the robust behavioral equivalence of any RNN to an FFN; see (Usher, 2021), this premise can be replaced with a simulation version (which is somewhat less controversial), but leads to an equally puzzling conclusion (see China Brain). Second, as the debate about P2 is somewhat technical^13^, we focus here on the philosophical/methodological premises that are more central to the theme of this research topic.

A number of philosophical and methodological criticisms of the UA were also put forward (Albantakis, 2020a; Negro, 2020; Tsuchiya et al., 2020), raising the objection that the UA ignores the relevance of first-person experience in consciousness research (P1). After all, if we want to account for the relation between phenomenal experiences and brain processes, “how it feels to be a conscious subject” is the property of interest (Nagel, 1974). Though eventually interested in phenomenal consciousness, many researchers prefer not to tackle directly phenomenal consciousness, and focus instead on functional aspects, such as *access* [see (Block, 1995, 2011; Dehaene, 2014)]. This risks neglecting what many believe to be the real explanatory target (and the most challenging and fascinating aspect) of consciousness science (Chalmers, 1995; Block, 2002; Ellia et al., 2021).

First, the UA prescribes a methodology for the science of consciousness that excludes the use of first-person data (P1). Instead, Herzog et al. (2022) insist that publicly available data (i.e., objective measurements usually coming from experimental results of consciousness experiments) must be the only source of evidence for consciousness science. According to them, this methodology does not dismiss first-person phenomenological observations, but requires “transforming” them into public data via introspective reports. Only at that point can first-person data, transformed into behavioral evidence, be mapped onto neural evidence. Second, while the UA does not preclude brain measurements in consciousness research, it prescribes such measurements to be carried out only in a second stage (P4), once the conditions for presence of consciousness are established exclusively by behavioral reports (P3-P4; Doerig et al., 2019; Herzog et al., 2022). According to P4, relying on neural measurements to determine consciousness properties leads to *circularity.*

Here, we argue that this methodology, which excludes first-person data, or requires re-interpreting them as third-person data for consciousness science, and which defers neural data to a later stage, is overly *restrictive*, at least when we focus on human-consciousness (we defer to the Discussion section for a distinction between consciousness in humans and in general). Obviously, neither side of the debate denies the importance of behavioral data, nor the importance of neural data. The disagreement stems from how much evidential weight different scholars put on different types of data, and particularly on phenomenological (i.e., first-person) data. While the UA proponents claim that input–output (i.e., behavioral) data are the primary evidence for consciousness science and that purely phenomenological data, which are not translated into some public marker, are not scientific data at all, we maintain that behavioral data like introspective reports can only be valuable heuristics to be used in experimental settings, but they cannot be taken at face value. Furthermore, we highlight that the validity of third-person methods in consciousness science is grounded on first-person data to begin with (see also Ellia et al., 2021).

To illustrate this point, we can resort to the inferences we are licensed to draw in no-report paradigms^14^ (Tsuchiya et al., 2015; Koch et al., 2016); see also (Overgaard and Fazekas, 2016) and (Block, 2019) for discussions. In their reply to Tsuchiya et al. (2020), Herzog et al. (2022) argued that even in these cases, we associate the presence of a conscious state with some sort of public measurement, such as optokinetic nystagmus or other physiological measurements. However, this is not always necessary. Suppose we present a non-masked, isolated, supra-threshold stimulus to an awake subject who attends to it. In that case, we can reasonably infer the subject will be conscious of the stimulus without relying on any sort of public measurement. The justification for this inference is provided by the fact that we are certain that *we* would be conscious of the stimulus in that condition, had we been in the subject’s place. This inference is facilitated by the assumption that our brain is similar enough so that we should end up with similar visual experiences when viewing the same object under similar conditions. Thus, first-person data can guide and constrain our inferences about other people’s experiences, and in this sense, they constitute an indispensable tool for consciousness research. This does not mean that first-person data must be the only evidence for consciousness science. In itself, the fact that an awake subject is conscious of an unmasked supra-threshold stimulus provides limited information on how consciousness relates to brain processes. But the no-report paradigms can be used to disentangle neural correlates of consciousness from that of reports and to eliminate confounding factors related to post-perceptual processes underpinning cognitive accessibility (Block, 2019; Malach, 2022). In fact, the reliance on phenomenal experience was one of the essential ingredients of the method of early psychophysicists (e.g., Fechner), who aimed to uncover laws that map the relation between the intensity of subjective experiences and objective aspects of the environment (see Ellia et al., 2021).

The role of first-person data for consciousness science can also be appreciated by focusing on the inferential reasoning that justifies our attribution of consciousness to subjects in standard experimental settings: we infer that a subject is conscious of the stimulus because we have a series of observations (e.g., the stimulus presentation, the subject looking at the screen, and so on) and some background knowledge that links those observations to consciousness. But crucially, if the information that *I* would be conscious of the stimulus if *I* were in the subject’s position was not part of that background knowledge, I would not be justified in inferring that the subject is conscious of the stimulus. Thus, first-person experiences can provide part of the justificatory ground for attributing conscious states to other people. In experimental settings, they can be used to justify inferences about the conscious states of tested subjects.

So far, we have focused on no-report paradigms to show that “first-person experience,” not transformed into public data/report, is relevant to consciousness science even when behavioral evidence is scarce or non-available. This means that first-person data do not necessarily need to be “transformed” into publicly available data to be of any use to consciousness science: even as phenomenological data, they are legitimate scientific tools^15^. We thus claim that verbal/behavioral reports (which are considered primary in the UA-rationale), only provide us with reasonable evidence of (first-person) experience via an inference to the best explanation, or via analogy, under “*appropriate-circumstances*.” We believe that minimal conditions of such “appropriate circumstances” should include the following epistemological conditions:

- We have no grounds to suspect that the person is lying or concealing information.
- The mechanisms connecting experience to output systems must be reliable (i.e., not “damaged” - so that they can relay experience).
- We know that the system is similar to us along relevant dimensions, like brain structures and information processes.

Condition (i) is violated if we have grounds to suspect that a person conceals information. In such a case we are justified not to take her verbal introspection reports at face value. Condition (ii) is violated in certain disorders of consciousness, when a person can be conscious, but unable to express their conscious states in the form of outputs because the output pathways are damaged (Owen et al., 2006; Monti, 2015).

Here, we focus on condition (iii), which allows us to see an important shortcoming of the UA. The difference between the FF-robot and the RNN-robot is that the latter is similar to humans, along the dimension of the brain causal structure, whereas the former is not. Moreover, we know that human brain structures that implement FF-like computation, like the cerebellum, have no particular role in constituting human consciousness (Massimini and Tononi, 2018).

Similarity with humans is important because human beings are the only creatures whose consciousness we are certain of. Indeed, Albantakis (2020a) argues that based on inference to the best (or at least, good enough) explanation, we have little reason to maintain that the FF-robot is conscious. This is because scientific investigation on human consciousness provides evidence that recurrence is necessary for human consciousness (Lamme and Roelfsema, 2000; Pitts et al., 2014; Koch et al., 2016) while there is no evidence of FF structures being relevant to human consciousness. Accordingly, we are licensed to put lower credence in the hypothesis that the FF-robot is conscious because it coheres poorly with neuroscientific background knowledge, derived from studies of *human* consciousness (in the Discussion section we will focus on what this stance implies for what we believe to be the *scope* of consciousness research).

To illustrate how this approach, which combines behavioral with phenomenological and neural data, is not circular (and also falsifiable), we apply it to testing the recurrence-hypothesis in human consciousness. This hypothesis, endorsed by IIT (Albantakis et al., 2023) and RP theorists (Lamme, 2006) and denied by the UA authors, states that recurrent processing is necessary for human consciousness. According to P4, making consciousness dependent on neural measures such as recurrent processes is circular. Here, we follow Kleiner (2020) and Kleiner and Hoel (2021) in arguing that using neural markers of consciousness does not require endorsing the theory we aim to test. For example, a theory of human consciousness may predict that a certain type of causal network in a particular state corresponds to a particular conscious experience. We can now carry out experiments to test such predictions by applying TMS (or some other intervention, say, optogenetics or pharmacology) that transiently stimulates/disrupts the recurrent processes in a specific brain area (see (Michel and Malach, 2022) for a similar idea). We do not need to rely on that theory again to interpret the predicted effect. Instead, we carry the experiments on ourselves (or on other volunteers) to determine if we *feel* a difference in experience [for the volunteers, we can rely on their introspective reports, provided that the above-mentioned appropriate circumstances occur; see also (Ruffini et al., 2022)]. Obviously, such experiments, ideally employing a multitude of methods, can provide converging evidence that either increases or decreases our degree of belief in the hypothesis under study (note that we qualify this to human consciousness), and we do not need to assume a theory to arrive at this result. In this scenario, we can either diminish or increase support for the claim that recurrence is necessary for human consciousness.^16^ So, the charge of circularity does not apply.

Finally, we wish to suggest a more ecumenical methodology for consciousness science, which acknowledges the need to integrate different types of data instead of relying solely on input–output data as primary source of consciousness data. These can be neuroscientific data, psychological data, and phenomenological data. For example, Block (2007) suggests considering both psychological and neuroscientific evidence. Similarly, Bayne and Shea (2020) suggest a natural kind strategy that is aimed to identify consciousness through a set of markers that cluster together: the scientific study of phenomena like hepatitis has improved by starting from a cluster of symptoms (e.g., fever, jaundice, etc.) to then investigate the biological underpinnings of the cluster (e.g., the presence of some viruses). In the same way, according to Bayne and Shea, consciousness science could take advantage of a cluster of observed “consciousness symptoms” to investigate the cluster’s mechanistic underpinnings. Our proposed “integrative approach” to consciousness science suggests that consciousness could be associated with a cluster of phenomenological, functional, and neural properties, and therefore evidence must be gathered from paradigms that are sensitive to all these properties [see also (Shea, 2012; Birch, 2022)]. This is not a very new idea, and is consistent with the fact that we often use brain measurements (e.g., polygraph) to validate the veridical status of verbal reports [see also (Koch and Tsuchiya, 2007; Block 2008) for a discussion of possible visual experiences in neglect patients]. Here, we stress the importance of including phenomenological data in this “integrative approach.” More specifically, phenomenological data can constrain the inferences allowed by observing neural and behavioral data, in the sense that they can define the legitimacy of those data for consciousness studies. In other words, without first-person data, we would not be able to explain why neural and functional data should be data about consciousness. The methodology for consciousness science we propose is thus a methodology that requires an integration of different types of data, and, contrary to the methodology suggested by the UA authors, acknowledges the necessary role of first-person experience in theorizing about consciousness.

It could be argued that any methodology founded on first-person experience is founded on shaky grounds, since we are prone to introspective errors and we are often confused about our experiences (Dennett, 1990; Cohen and Dennett, 2011; Schwitzgebel, 2011) - for a discussion, see (Smithies, 2013). However, our methodology does not rely on the assumption that our beliefs of what we are conscious of must be accurate, but only that we have some sort of phenomenological awareness of the contents of consciousness. The awareness itself, and not what the awareness is about, is what constrains our theorizing about consciousness, and it is thus the foundational datum for the science of consciousness. As Searle puts it, “consciousness consists in the appearances themselves. *Where appearance is concerned we cannot make any appearance-reality distinction because the appearance is the reality*” (Searle, 2008, p. 76; italics in the original). Thus, we believe that a fundamental mistake implicit in P1, P3, P4 is the assumption that behavioral data is primary to consciousness studies [see also (Kleiner, 2020; Kleiner and Hoel, 2021)].

In addition, we also believe that assumptions P1, P3, and P4 are overly restrictive, in virtue of a verificationist approach to science, which considers as scientifically meaningful only those statements that can be empirically verified. Philosophers of science have pointed out that this approach implies a clear distinction between empirical and theoretical statements, which is often unwarranted (Quine, 1951; Hanson, 1965). Surpassing this restrictive verificationist stance can ensure that other important aspects of scientific theories, like parsimony (as measured by complexity measures), consistency with background knowledge, and elegance be included in the practice of consciousness science, and there is no need to fear statements that are not directly verifiable: the scientific status of theories does not depend on whether they are constituted uniquely by empirically verifiable statements, but depends instead on whether the type of research program they generate is progressive or not (Lakatos, 1970).^17^

To conclude, we believe that in consciousness research we need to start from our own phenomenal conscious experience as primary, and investigate its physical underpinning, to be searched in the neural data. Behavior, of course, should be used, but may not always be needed (when our conscious phenomenology is clear enough, for example).

## 6 Discussion

We have reviewed the UA argument against causal structure theories of consciousness. We argued that the argument rests on multiple assumptions that are either not formally proven or reflect a set of overly restrictive philosophical assumptions about the proper methodology of consciousness research. We have also argued that if the rationale of the UA argument is accepted, one can construct a similar argument that targets not only causal structure theories but also functionalist ones [see also (Kleiner and Hoel, 2021)]. We believe this is the outcome of the UA-assumptions, which, despite the authors’ aspirations, make functionalist theories of consciousness scientifically illegitimate and leaves little logical space for theories of consciousness.

We have suggested that premises P1, P3, and P4 are too restrictive, methodologically speaking. Instead, we propose an integrative approach, in which consciousness can be studied in tandem, through phenomenal, behavioral and neural data (Bayne and Shea, 2020). In particular, we have suggested that similarities in brain processes and structures are crucial to determine the presence and types of conscious states. Below we highlight several implications that this approach to consciousness research has, and we discuss some potential objections.

### 6.1 Restrictive methodology vs. restriction on the scope of current consciousness research

According to the UA, consciousness science should be primarily based on behavioral data – purely first-personal observations and ‘direct’ brain-based evidence (unmediated by behavior) are excluded, and a large set of theories are false or lie outside the realm of scientific investigation. However, since for many of us phenomenality, as grasped from the first-person perspective, is the primary aspect of consciousness (i.e., phenomenal realism; see (Block, 2002)), this seems equivalent to proclaiming that there can be no science of consciousness. Here we propose a different kind of limitation on consciousness research – one on the range of systems upon which theories of consciousness should be tested and built. Specifically, we argue that, at least at this stage, theories of consciousness should be tested and built upon the case of human consciousness.^18^ But this restriction on the range of systems that can test theories of consciousness does not imply a methodological limitation: we can, and should, be bold concerning the methods we employ to study human consciousness, giving pride of place to first-person and brain-based evidence.

We argue that the distinction between these two types of restrictions is crucial to theoretical perspectives on consciousness research in general and to the UA in particular. In fact, our proposed limitation concerns the domain of theory-testing (i.e., how we test a theory against empirical data), whereas the “hard criteria” suggested by Doerig et al. (2021) concerns theory-building (i.e., how a scientific theory is constructed in the first place) (for specific criticisms of their criteria, see Fahrenfort and van Gaal, 2021; Haun and Tsuchiya, 2021; Seth and Hohwy, 2021). Thus, we propose that, as far as consciousness research in humans is concerned, the restrictions expressed by premises 1 and 4 of the UA, and hence its conclusion, should be rejected. Specifically, in this case, contra P1, consciousness science need not rely *only* on physical measurements (like behavioral data), as in the no-report paradigm and, contra P4, it can use direct measurements of brain activity (independently of behavior). This is because, first, we all know, from first-person experience, that there are conditions when we are conscious of a suprathreshold object on display without distraction. Second, the deep similarity to other humans makes the generalization possible (Sober, 2000).

Things are different concerning the extrapolation to non-human consciousness (in cases where there is no significant similarity with human ‘hardware’). In this case, only behavioral measurements are available and direct measurements of ‘brain’ (or other ‘hardware’) activity are of little use to the scientist. But this is because (in this case) we lack the first-person perspective from the very outset. And in the complete absence of similarity to humans, extrapolation becomes more difficult.

The limitations on the investigation of non-human consciousness are reflected by various familiar philosophical lines of reasoning, such as Ned Block’s *“harder problem of consciousness*” (Block, 2002), which argues that we lack rational ground for believing that systems that do not share our physical realization are or are not conscious. Consider Commander Data^19^ – a robot whose functional organization is similar to that of a human but whose physical realization is quite different. *Prima facie*, the functional similarity seems to provide a reason for attributing consciousness to Data, yet, the physical dissimilarity seems to provide a reason for denying such attribution. On the one hand, upon interacting with Data, you will likely take it for granted that he is conscious. On the other hand, upon discovering that he is a robot with a different ‘brain’ realization, your intuition might be that he is non-conscious. Block argues that there is no rational ground for adjudicating between these intuitions. We have no conception of rational belief to the effect that Data *is* or *is not* conscious – Data’s consciousness is meta-inaccessible: “Not only do we lack a ground of belief, but we lack a conception of any ground of belief” (Block, 2002, p. 405). According to Block, the deep root of this epistemic problem is that we lack the justificatory basis to generalize the science of consciousness to systems like Commander Data.

"the example of a conscious creature on which the science of consciousness is inevitably based is us […] But how can science based on us generalize to creatures that do not share our physical properties? It would seem that a form of physicalism that could embrace other creatures would have to be based at least in part on them in the first place, but that cannot be done unless we already know whether they are conscious" (Block, 2002, p. 407).

The problem, then, is not that the first-person perspective (independently of behavior) is not crucial for the study of consciousness, but that we lack that perspective in the case of Commander Data and other differently realized creatures. The same rationale holds with respect to the FF-robot discussed by the UA.

The idea that we can learn about consciousness, in general, from what we know about human consciousness, specifically, is problematic. Since it is unclear whether we can directly use our knowledge of human consciousness as justificatory ground for the attribution of consciousness to entities significantly different from us along various dimensions, it is not clear that we have a justificatory basis to either exclude or include the FF-robot from the realm of conscious entities. This would remain true even if we ascertain that in the human case consciousness depends on some kind of causal structural properties. Such confirmation of particular causal structural properties may not be necessary for consciousness in other systems. Note, though, that neither can we know whether functionalist theories capture what is crucial for non-human consciousness, since a functional organization similar to our own may be neither necessary nor sufficient for non-human consciousness. Thus, the epistemic problem that concerns extrapolation to other (differently realized) minds afflicts not only theories of physical realization (and causal structure theories), but also theories of functional organization. Earlier, we argued that in the human case, first-person and (‘direct’) neurological data are available, so all levels of inquiry and all theories of consciousness are legitimate. Our present point is that in the case of alien consciousness, the relevant kinds of data are unavailable and the rationale guiding our (human) consciousness-theories is inapplicable.

Given this situation, we argue that there are two theoretical options. The first is to adopt a “humility principle”: given the human-centered methodology for consciousness science we are advocating, at this stage, we should in fact remain silent on alien, or non-human, consciousness. If the humility principle is adopted, the results of consciousness science should not generalize beyond creatures that are relevantly and substantially similar to us (see (Carruthers, 2019) for arguments supporting a similar position). This does not mean that we should not care about non-human consciousness, or that non-human consciousness will forever remain beyond our reach. Rather, the humility principle warns us that the current knowledge we have about consciousness is highly context-dependent (i.e., based on human-consciousness), and therefore many inferences currently drawn about non-human systems [whether they are theory-driven or not, see (Birch, 2022) for a discussion and (Butlin et al., 2023) for a case-study] might be unwarranted.

The second option is to adopt a more ambitious stance, by either formulating theories in a context-independent way [this is what Kanai and Fujisawa (2023) call ‘universality’] or by justifying extrapolations to the non-human case through arguments based on analogical reasoning, abductive reasoning, or a combination of both (Melnyk, 1994). One could start, for example, from the structural similarities between the source domain (i.e., the domain for which the original hypothesis is formed, for example the domain of humans in the case of consciousness) and the target domain (i.e., the domain for which the hypothesis is supposed to hold, for example organisms radically different from humans and non-biological systems in the case of consciousness), and then claim that given that phenomenal properties correlate with specific properties in the source domain, the most parsimonious hypothesis is that a correlation between similar properties in the target and phenomenal properties occurs (Barron and Klein, 2016; Godfrey-Smith, 2017; Bayne and Shea, 2020; Tsuchiya and Saigo, 2021; Birch, 2022). In this case, inferences about the conscious state of target systems could be justified, thus solving Block’s ‘harder-problem’ (Hohwy, 2004), by acknowledging how often in science unobservable entities are legitimately posited in the context of discovery (for example, the electron). These inferences can be conjectures driving further testing, and if they are based on brain (or at least, “hardware”) similarity, they could potentially be tested by implanting specific structures, mimicking the relevant structures of target systems, into our own brains [see also (Shevlin, 2021) for a discussion].

These two options have contrasting strengths and weaknesses: the humility principle can ensure that our applications of consciousness theories are more grounded, at the cost of limiting the explanatory power of such theories. Adopting the more ambitious stance, instead, can ensure stronger explanatory power, at the cost of either requiring a further ampliative argument or formulating theories that might end up being too liberal in their ascription of consciousness [for discussions, see (Block, 1978; Block, 2002; Shevlin, 2021; Kanai and Fujisawa, 2023)].

Independently of which of these two stances toward extrapolative practices is favored, we argue that consciousness science should be firmly built on evidence gathered from humans (Negro, 2020), and such evidence should include not only behavioral evidence but also neural and phenomenological (first-person) evidence. This means that human-based theories of consciousness should not be dismissed because of what they predict about non-human consciousness (Albantakis, 2020a; Tsuchiya et al., 2020).

### 6.2 Objections

#### 6.2.1 Non-circular testing of the recurrence hypothesis

In section 5.1, we have argued that it is possible to test, in a non-circular way, the hypothesis that recurrence is necessary for human consciousness. One objection that we anticipate to the TMS-test we propose is that one should not just interfere with recurrent processes, but replace them with some appropriately tuned FF-circuits. Let us assume that such a circuit exists, that allows an FF-robot to respond as a normal human in consciousness experiments (it responds that orange is more similar to red than yellow, that this pain is unpleasant or that this stimulus has not been seen). Obviously, there is no way to test this robot in our experiment which affects recurrent connections (as it does not have any). This could be a problem if we aim to establish a general theory of consciousness, that extends to non-human C, because if the FF-robot is in fact conscious, then the hypothesis that recurrence is necessary for consciousness *in general* would be disproved. So, if the goal were to build a theory of consciousness in general, we would require a further argument to show that the FF circuit is not consciousness-generating in general, and not only in humans. But if we adopt the humility principle we can restrict the scope of the TMS-experiment above-proposed, and safely conclude that it offers a critical test of human-C,^20^ whereas if we maintain the possibility of extrapolation to non-human systems, we need an argument to show that the FF-circuit is not conscious. Thus, this objection could put pressure on the generality of the scope of consciousness science for those who do not subscribe to the humility principle. Still, it does not seem decisive against the idea that the TMS-test we proposed is valid for testing the recurrence hypothesis in humans.

## 7 Conclusion

To conclude, while the UA has opened a stimulating debate that contributed to clarifying a number of conflicting intuitions on the nature of consciousness^21^, we believe that the (hard-criteria) UA program (Doerig et al., 2021) is too restrictive and that it hinders, rather than promotes, the scientific research of consciousness. We identified several problems, involving both philosophical and methodological viewpoints and proposed an alternative less restrictive approach that facilitates the convergence from phenomenology, theory, and neuroscience. As consciousness research is primarily based on phenomenology in humans, we cannot directly access non-human consciousness. In turn, this means that conjecture about (potential) non-human consciousness cannot be used to restrict a class of consciousness theories as the UA attempted.

## Data availability statement

The original contributions presented in the study are included in the article/supplementary material, further inquiries can be directed to the corresponding author/s.

## Author contributions

MU: Conceptualization, Methodology, Supervision, Writing – original draft, Writing – review & editing. NN: Conceptualization, Methodology, Writing – review & editing. HJ: Conceptualization, Methodology, Supervision, Writing – review & editing. NT: Conceptualization, Methodology, Supervision, Writing – review & editing.

## Footnotes

^1^Most present-day dualists adhere to property (rather than substance) dualism, according to which mental properties exist “over and above” physical properties. An important distinction is between interactionist dualism and epiphenomenalism. *Interactionist dualism* (Descartes, 1641/1996) comes under conflict with the entrenched principle of the *causal closure of the physical law*s. *Epiphenomenalism,* on the other hand, is unattractive because, among other things, it does not allow for an evolutionary account of consciousness (Braddon-Mitchell and Jackson, 2007).

^2^Although this passage focuses on logical behaviorism, both logical and methodological variants of behaviorism are at odds with functionalism, with respect to how mental states are determined.

^3^One may consider cognitive psychology as a very successful functionalist project, which transcended methodological behaviorism and folk-psychology, by relying on experimental manipulations of sensory inputs and observing behaviors (including reaction-time and eye-movements) to infer internal states and processes, such as memory, goals and attention processes, or cognitive inferences, which mediate complex behaviors.

^4^Note that while Doerig et al. (2019) focused particularly on IIT, the UA targets all *causal-structure* theories and not only IIT. We also wish to clarify that we are agnostic on whether IIT and RPT are successful theories or not. We hold, however, that they are both valid hypotheses that should not be dismissed without empirical tests, on purely theoretical grounds.

^5^This needs to be qualified in relation to the distinction between substrate and network invariance. Causal structure theories (like IIT and RPT) might be considered as functionalist from the perspective of substrate-invariance, but not from the perspective of network-invariance as they hold that consciousness depends on the network structure but not on its material composition (e.g., carbon vs. silicon). One potential interpretation of causal structure theories is that they require algorithmic rather than implementational recurrence (Butlin et al., 2023, p 21). However, this faces the problem that the algorithmic level is just not well defined, in general. The assumption that neurons count as the level of implementation is adhoc. More specifically, IIT requires network (rather than algorithmic) recurrence, which might still be seen as a functional property but at a higher resolution that just behavior (see next section).

^6^Herzog et al. (2022) state that “A behaviorist would claim that internal states are useless to understand the mind, if they would use the word “mind” at all […]. In contrast, we take subjective states seriously and assume that we can learn about them through i/o observations. We cannot measure consciousness directly, but we can measure subjective reports (verbal or otherwise) and link them to brain activity” (p. 3).

^7^More precisely, this conclusion applies to any model of consciousness that depends non-trivially on physical systems [see definition 2.8, and Lemma 2.9, in Kleiner, (2020)].

^8^This tripartite distinction is inspired by Marr’s three levels of analysis Marr (1982). Marr called level one "the computational level", but his characterization of this level makes it clear that it is concerned with the abstract specification of behavior. As Love (2015) puts it: "The nature of the computing device (i.e., implementation level) and how the computation is carried out (i.e., the algorithmic level) are irrelevant at this level of analysis. The sole concern of Marr’s computational level is the abstract problem description, which consists of detailing the input–output relationships.

^9^We believe that both of these statements need to be qualified. The indeterminacies above are likely to apply when one examines simple functions from one space to another, *Y* = f (X) (say, like in a categorization task that requires subjects to name visual objects). It is more doubtful that they apply in the case in which the transformation applies to temporal entities, which impose restrictions on the temporal duration of the transformation.

^10^To mention but two simple examples, the *Quick-Sort* algorithm applies a divide and conquer strategy to divide a list into sub-lists: pick an element, called a pivot, from the list. Reorder the list: locating all elements with values less than the pivot before the pivot and all elements with values greater than the pivot after it (equal values can go either way). The sub-arrays are then sorted recursively. The even simpler *Bubble-Sort* algorithm, in contrast, works by repeatedly stepping through the list, comparing each pair of adjacent items, and swapping them if they are in the wrong order, until no swaps are needed.

^11^If one considers a program that computes x + n-n rather than x as a different algorithm, then we have a proof already. However, one may object that an optimizing compiler, which operates on this program, would replace the line x + n-n by just x. The question can thus be reformulated as: “Do we have for each function, a single algorithm that cannot be reduced by any optimizing compiler to the same program (i.e., which are not equivalent in any sense other that they represent the same function)?.” This question can be expressed by Turing machines. There are two important results: (i) For every Turing machine there is an equivalent (but not identical) Turing machine; (ii) There is no program that can decide whether any two Turing machines are equivalent. Therefore, as a compiler is a program itself, it is not possible that the compiler can reduce one program of the function to another one, or recognize them as equivalent, for any two equivalent programs [for a review, see (Miller, 2014)].

^12^This is because for any algorithm that underlies conscious behavior one may construct a behaviorally equivalent different algorithm (like in Blockhead), and then argue that attributing consciousness to one but not the other is either false or untestable. Contrary to what Herzog et al. (2022) claim, there are mathematical theorems showing there are multiple algorithms that can mediate the same input output function.

^13^To our understanding the UA concludes that *since both FFN and RN are universal approximators they can approximate each other.* We believe that this conclusion is based on a logical fallacy. From the fact that FFN and RN are approximators of different entities (an FFN approximates functions, while an RN approximates dynamical systems), it does not follow that they approximate each other. First, the output of an RN cannot be characterized as a function of its input alone (which is what FFNs can achieve), since it also depends on the state of the network itself. This is a critical property of RNs, allowing them to exhibit dynamic properties such as self-sustained activation states without any input and to manifest the dynamical property of hysteresis. This is particularly important for our aims, because many conscious states (e.g., dreams) depend on this sort of self-sustained activation states that are possible only for RNs, and thus appear (in the absence of input) outside the reach of FFNs. Second, there are independent reasons to reject P2: research within the field of neural computing (Siegelmann and Sontag, 1995; Cabessa and Siegelmann, 2012) indicates that FFN and RN differ drastically in terms of their computational power: while the former are far below Turing-computation, the latter can exceed it (see (Ruffini et al., 2022) for a recent and detailed discussion; see also Usher (2021) for an illustration showing that apparently equivalent RN and FFN, are not equivalent when tested with perturbations, and replies by Herzog et al., 2022).

^14^In such paradigms the conscious status of the subject is inferred without requiring a verbal report (Tsuchiya et al., 2015; Koch et al., 2016). These are paradigms that seek to distil the confounds of neural correlates of reports from the true correlates of phenomenal consciousness (Aru et al., 2012). For example, a no-report design for binocular rivalry replaces the verbal report with the monitoring of the optokinetic nystagmus (that tracks the eyes’ movement).

^15^We acknowledge the existence of views according to which there is no reality to phenomenal experience, beyond what is expressed in overt judgments or in intentional content (Dennett, 1990). In this paper we address only views that accept the reality of conscious experience (see also Ellia et al., 2021).

^16^The test suggested above examines the necessity of neural recurrence to consciousness. A similar test can be suggested to test its sufficiency. In the latter, one starts from a no consciousness state (say, a dreamless sleep) and carries out an intervention that enhances the strength of recurrent connections. If the subject experiences an increase in consciousness (say transition to dream state) this would provide support for the idea the neural recurrence is sufficient for consciousness. It is not our aim here to argue for either the necessity or sufficiency of recurrent activity, but only to suggest that they are valid scientific hypotheses.

^17^Contrary to what the strongest form of verificationism implies, there are abundant examples showing that directly unobservable entities (atoms, electrons or black holes) are taken as real in the physical sciences, once they enable us to account for a variety of data in a parsimonious way. This makes them valid scientific entities (for example, Boltzmann atomic theory was accepted despite strong opposition from Ernst Mach, based on positivist/verificationist commitments). The phenomenal experience of others is a similar aspect of reality we believe science needs to accept as valid, rather than restricting itself to verbal/behavioral protocol. We acknowledge that the UA proponents might consider phenomenal experience as a scientifically valid construal that is inferred from observations, rather than being directly observable. However, although that might be true for other systems, consciousness remains directly observable in us. If UA proponents do not want to endorse the strongest form of verificationism, they would need to accept the scientific legitimacy of the first-person perspective.

^18^In fact, “human-consciousness” might be too restrictive: biological systems which are neurologically similar to humans may also fall under the explanandum of current consciousness research. The central point is that systems that are very different from humans in their internal structures, such as FF-robots, indeed fall outside the scope of current science.

^19^Commander Data is a fictional character in the *Star Trek* franchise.

^20^We wish to clarify that the aim of this test is not to select between a functionalist and a brain identity theory based on a specific neural mechanism. We rather accept that the neural mechanism of consciousness has functional characteristics (such as enhancing perceptual discriminations and motor control), thus we fully expect that a disruption of the consciousness mechanism has functional effects. The test only asks if the neural mechanism has a causal (or constitutive) relation to consciousness in humans.

^21^See Gidon et al. (2022) for a recent thought experiment involving replay of neural activity associated with consciousness, which further probes some striking predictions of causal-structure theories of consciousness. The replay is not only guaranteed to generate the same responses to the same inputs (as in UA), but also enforces the same spike trains in all neurons.
